# Tuberculosis, human rights, and law reform: Addressing the lack of progress in the global tuberculosis response

**DOI:** 10.1371/journal.pmed.1003324

**Published:** 2020-10-23

**Authors:** Matthew M. Kavanagh, Lawrence O. Gostin, John Stephens

**Affiliations:** 1 Department of International Health, Georgetown University, Washington, DC, United States of America; 2 O’Neill Institute for National & Global Health Law, Georgetown University, Washington, DC, United States of America

## Abstract

Mathew Kavanagh and co-authors discuss law reform in the global tuberculosis response.

**Summary points**

- Tuberculosis (TB) remains the world’s leading infectious killer with 10 million annual cases and only limited progress in recent years when it comes to reducing new cases. Progress is hampered by continuing gaps in detection and treatment.
- With evidence that TB programs are hampered by the legal environment, we analyze the degree to which law reform is needed to align with promises at the United National High Level Meeting on Tuberculosis to promote human rights and dignity in the TB response.
- Exploring the laws at key points of vulnerability to TB in 20 high TB-burden countries (HBCs) shows that internationally recognized rights related to medical isolation, privacy, home inspection, and medical examination are insufficiently protected in the laws of most of these countries.
- Exploration of the legal environment for migration shows that restrictions or bans on travel and immigration for those with TB is the norm, along with limitations on access to medical care, in contravention of international standards.
- Fundamental human rights, expected in other areas of public life, are often missing for people with TB. This is likely contributing to reluctance among people with TB to engage with health systems. Law reform is urgently needed to align national laws with global human rights agreements and public health standards and help close major gaps in TB diagnosis and treatment.

In 2018, the United Nations General Assembly convened the first-ever high-level meeting (HLM) on tuberculosis (TB). Since that time news on the world’s most lethal infectious disease is not good—the 2019 WHO TB report shows 1.2 million people died from TB, a number that has fallen just 11% since 2015, less than one-third of the way towards the End TB Strategy milestone of a 35% reduction (to about 850 million deaths) by 2020. The same number of people, 10.0 million, are estimated to have fallen ill with TB in 2018 as in 2017 [1]. The stubborn persistence of TB is attributable to glaring gaps in case detection and treatment. While case detection has increased in recent years, there is still a significant gap between the 7 million new cases reported and the 10 million incident cases estimated in the most recent WHO data—and treatment success continues to hover at only 85% [1].

There has long been a call for a shift toward a “human rights-based approach” in TB—building policies and programs explicitly on the norms and values set out in international human rights law, treating people with TB as rights holders in their interaction with the state and the health system, and working to overcome stigma and discrimination [2–5]. Yet laws in high TB-burden countries (HBCs) have not been reformed to include basic rights protections. This helps explain the lack of progress, as individuals who fear discrimination and coercion may avoid diagnosis and treatment [6,7]. Thus, TB stands in stark contrast to HIV/AIDS, for which the international community has moved far more decisively to recognize and protect key rights, even as more progress is needed [8,9]. At the UN, heads of state committed to removing discriminatory laws and policies against people with TB, protecting and promoting their human rights and dignity, as a key part of a strategy to improve the TB response and end the epidemic [10]. Here, we analyze key areas of law with particular importance for TB, focusing on five aspects of compulsory public health powers in law and on laws related to migration. We identify significant gaps between core human rights norms and existing legal environments.

## TB-related public health laws

To better understand one facet of how TB and the law interact, we analyzed laws relating to compulsory public health powers of the state in 20 HBCs, evaluating whether core human rights protections were built into public health laws used in TB cases (Table 1). We used Westlaw, Lexus Nexus, and Google searches in January 2019 and repeated in December 2019 along with a review of Legal Environment Assessments conducted by the Stop TB Partnership [11] to identify the national laws governing these areas. Additional legal text was gathered through personal requests to legal and TB experts in countries where our efforts had not yet yielded appropriate legal text. In all, we were able to acquire full legal text for 20 of the 30 HBCs identified by the World Health Organization (WHO) [12]. These are analyzed below, using and augmenting data collected for a policy briefing for member states at the UN High Level Meeting [13]. Based on legal tradition and custom, countries differ on how they deal with TB in law—some include explicit mention of TB or even TB-specific laws, while others are governed by more general law related to infectious disease. Our review encompasses TB-specific laws and, where absent, broader applicable infectious disease legislation. In federalist systems, unless otherwise specified, the review centers on national-level legislation. We developed a set of five questions, each of which is described below, with which to code the laws of each country to compare national incorporation of key rights into law. WHO’s Ethics Guidance is one of the few international benchmarks available for appropriate norms related to TB, so it was used as a starting point for analysis, read in conjunction with international literature on health, ethics, and human rights [14–17].

**Table 1:** TB-related laws on isolation.

Country	1. Reasons for forced isolation: Does the country’s law include substantive protections that dictate when mandatory isolation can be used, in accordance with the World Health Organization Ethics Guidance for Implementation of the End TB Strategy?	2. Fair process in forced isolation: Does the country’s law include procedural protections including the right to notice and appeal or challenge when coercive (mandatory) measures are used, in accordance with the World Health Organization Ethics Guidance for Implementation of the End TB Strategy?
Bangladesh	** **	** **
Brazil	** **	** **
China	** **	** **
Ethiopia	** **	** **
India	** **	** **
Kenya	** **	** **
Lesotho	** **	** **
Liberia	** **	** **
Myanmar/Burma	** **	** **
Namibia	** **	** **
Pakistan	** **	** **
Papua New Guinea	** **	** **
Russia	** **	** **
Sierra Leone	** **	** **
South Africa	** **	** **
Tanzania	** **	** **
Thailand	** **	** **
Vietnam	** **	** **
Zambia	** **	** **
Zimbabwe	** **	** **

Red = no, yellow = partial, green = yes.

Additional HBCs are: Angola, Cambodia, Central African Republic, Congo, North Korea, Democratic Republic of Congo, Indonesia, Mozambique, Nigeria, and Philippines.

**Abbreviations:** HBCs, high TB-burden countries; TB, tuberculosis.

## Medical isolation

Governments have an obligation to rapidly detect and treat TB cases and to prevent exposure to uninfected individuals. Community-based and outpatient interventions have proven most effective in treating people with active, contagious TB [18,19], but compulsory isolation may sometimes be necessary. International human rights treaties, however, oblige states to avoid arbitrary or indefinite detention, affording individuals the right to due process of law [20,21]. The UN Economic and Social Council’s Siracusa Principles specify that deprivations of liberty must be evidence based and the least restrictive means. WHO guidance on TB states that governments should use coercion only when it is necessary, likely to be effective, when less-restrictive means have been rejected or are not feasible, and for the minimum period necessary [14].

Laws in many countries do not guarantee these basic rights protections. Shown in Table 1, indicator 1 codes whether a country’s law describes when and how health agencies can compel isolation. Since there is not a particular consensus agreement on exactly when isolation is necessary with regards to TB, we code simply whether laws indicate some criteria or mirror the language around “necessary,” which might be interpreted through local law. Countries are coded red when they do not contain clear criteria and green when they align with WHO guidance. Indicator 2 codes whether a country’s law includes basic due process—notice of why individuals are being detained, with a hearing before or soon after detention takes effect. A country is coded red if it provides for neither notice nor a hearing, yellow if it provides notice or a hearing, and green if it provides both. Taken together, Table 1 shows that national legislation most often does not guarantee even minimum rights—criteria delineating a clear public health justification and few, if any, procedural safeguards to empower individuals to defend or regain their liberty through a fair hearing. South Africa offers a rare illustration of laws that do meet international standards, clearly spelling out in its recently revised regulations that public health officials must consider “the need, nature and extent of the intervention, based on the nature of the public health threat and the particular circumstances of the individual” as well as the likelihood of transmission [22]. Involuntary isolation is only allowed after the person in question “has been offered and encouraged to accept counseling services” on voluntary cooperation and then only upon a court order, issued with clear notice and a chance to appeal the decision through administrative means.

Weak national laws, of course, do not suggest that all or most health officials act in arbitrary ways—only that national legal systems fail to offer procedural and substantive safeguards required under international law.

## Privacy, inspection, & examinations

Public health agencies have the power to inspect homes and workplaces to ensure they are not driving TB transmission. Many laws allow public health authorities to examine people in the inspected location. International agreements and domestic constitutions, meanwhile, afford a right to be free from arbitrary or unlawful interference in the family and home [20,21]. Nonconsensual searches must be based on objective evidence, subject to authorization by an impartial authority through a warrant or other means. Similarly, international and domestic laws protect the autonomy, security, and bodily integrity of the person, requiring reasonable cause and due process [23]. Reviewing laws in HBCs (Table 2), indicator 3 asks whether a country’s law requires evidence to justify nonconsensual inspection of homes and whether there must be independent authorization, such as through a warrant. A country is coded red if its legal framework requires neither an evidentiary basis nor independent authorization, yellow if it requires one or the other, and green if it requires both. Significant differences exist among countries coded yellow—some require an evidentiary basis to justify entry and search but set a low bar, such as “reason to believe” someone has, or has been exposed to, TB. These weak laws may not even require reasons to believe there is a risk of further transmission.

**Table 2:** TB-related laws on privacy.

Country	3. Arbitrary Entry & Inspection of Homes: Does the country’s law on public health inspections have procedural protections against arbitrary entry and inspection of homes?	4. Forced Medical Examination While Inspecting Homes: Does the country’s law on public health inspections have procedural protections against arbitrary use of mandatory medical examination of persons in inspected homes?	5. Reporting Obligations & Criminalization: Does the country’s law refrain from placing notification / reporting requirements on lay people and creating criminal or civil offenses for noncompliance?
Bangladesh	** **	** **	** **
Brazil	** **	** **	** **
China	** **	** **	** **
Ethiopia	** **	** **	** **
India	** **	** **	** **
Kenya	** **	** **	** **
Lesotho	** **	** **	** **
Liberia	** **	** **	** **
Myanmar/Burma	** **	** **	** **
Namibia	** **	** **	** **
Pakistan	** **	** **	** **
Papua New Guinea	** **	** **	** **
Russia	** **	** **	** **
Sierra Leone	** **	** **	** **
South Africa	** **	** **	** **
Tanzania	** **	** **	** **
Thailand	** **	** **	** **
Viet Nam	** **	** **	** **
Zambia	** **	** **	** **
Zimbabwe	** **	** **	** **

Red = no, yellow = partial, green = yes.

Gray indicates laws that could not be coded.

**Abbreviation:** TB, tuberculosis.

Indicator 4 asks whether authorities are also empowered to medically examine people within homes on the same basis that authorized the initial entry. Countries are coded red if the grounds for entry and physical examination are identical or green if their law requires some additional grounds or procedure. Note that most countries’ laws authorize mandatory medical examination in other contexts, but this indicator does not address those circumstances. Indicator 4 shows that few countries have legal safeguards to assure clear criteria and fair procedures as required in international law.

There is both diversity between countries in their alignment with human rights norms and often within countries’ laws. In Lesotho, for example, the law requires that entry into the home must be “necessary” for work “required or authorized by this Order” (Lesotho Public Health Order of 1970, Sec 87(1)), a standard that limits the powers of arbitrary inspection of homes, but does not include a protection for subsequently compelling medical exams for people in those homes who, for example, are suspected of having TB.

## Reporting and criminalization

An effective TB response requires a robust system to collect, analyze, and respond to data about the incidence, prevalence, and spread of the disease. Such systems often rely on health workers to report cases of TB to a public health agency. Some laws require all people—not just health workers—to report. Failure to report often carries a fine, even imprisonment. Thus, family members, teachers, employers, and neighbors may be under a duty to report, even if they have no training to identify TB cases. As a general principle of law, individuals should not be required to perform acts that they are incapable of effectively complying with. From a policy perspective, these laws may also instill a culture of secrecy, driving TB underground. In Table 2, countries are coded red if their law requires laypeople to report TB and renders the person liable to imprisonment for noncompliance, yellow if the offense is not punishable by imprisonment, and green if they do not require lay reporting or if a failure to comply is not an offence subject to penalty.

## TB, migration, and the law

**Fig 1:**
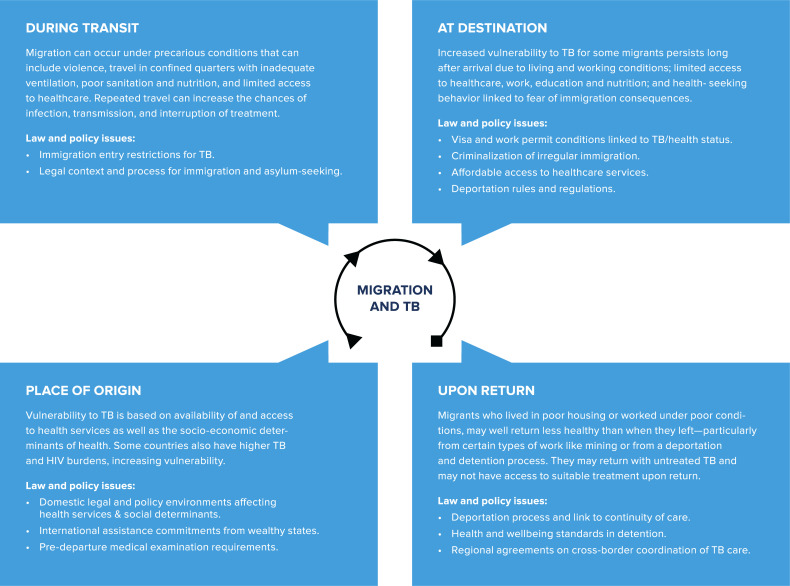
Migration & tuberculosis.

Mass international migration is greater today than at any point since World War II. WHO estimates there are 1 billion migrants, of whom 258 million are international migrants and 763 million are internally displaced—one in seven of the world’s population. Migrants—whether moving for economic opportunity, family reunification, or to flee conflict and humanitarian disasters—are at elevated risk of TB. Indeed, migrant populations represent a significant portion of TB epidemics in many contexts, though the relationship between migration and TB is complex [24–27]. Cambodian migrant workers deported from Thailand, for example, have TB rates almost four times greater than the general Cambodian population [28]. TB in foreign-born individuals is commonly the result of reactivation of latent infection acquired outside the host country. Transmission from migrants to the native population is often modest, yet migrants are nonetheless at significantly higher risk of the health effects of TB activation and transmission [29]. We looked beyond HBCs and considered migration more broadly—expanding our law review to review secondary literature and UN reports on the issue of migration and TB. Vulnerability to TB is heightened throughout the migration process (see Fig 1) due to the following: higher TB rates and weak health systems in countries of origin; unsanitary and overcrowded conditions during the journey; and exclusion from safe housing and social/health benefits in destination countries. Poor nutrition in each phase similarly increases risks [30–32]. Studies show migrants’ and refugees’ vulnerabilities persist long after they arrive at their destinations, due to poor working and living conditions and lack of access to care. For example, among Somali migrants and refugees living in Denmark, high TB incidence persisted for seven years after arrival [33]. Internal migrants in Shanghai, China, represent a large portion of TB cases, but more than two-thirds are infected after migrating [34]. Migrants returning to countries of origin, especially those engaged in work that subjects them to high TB risk (e.g., mining) are often not effectively equipped to prevent onward transmission to their communities of origin.

Yet despite complex vulnerabilities, many countries simply ban entry or residence—an attempt to keep TB out likely to be futile in a globalized world of extensive travel and limited ability to detect TB. During the height of the AIDS pandemic, many countries restricted the travel or immigration of persons living with HIV, which was widely condemned as ineffective and rights violating. A review of key literature and legal statutes shows that many countries are failing to heed this lesson and ban entry or residence of persons with TB, despite their complex vulnerabilities [35]. In the United States, active TB remains one of the seven listed diseases that triggers inadmissibility under the Immigration and Nationality Act, extending in some cases even to refugees [36]. Similarly, Canada identifies active TB as a condition “dangerous to public health,” rendering foreign nationals inadmissible on grounds of being a “danger to public safety” [37]. Australian, New Zealand, and United Kingdom law all condition entry for migrants on a negative TB diagnosis. These legal barriers are not limited to high-income countries. China precludes visas for foreigners with TB as well as for “other infectious diseases that may severely jeopardize the public health” [38]. In Liberia, the list of those who are “ineligible to receive visas and shall be excluded from admission… [include those] who are afflicted with tuberculosis in any form” [39].

Compounding the problem, some countries withhold essential medical services for migrants; others require people with TB to undergo treatment as a condition of legal status; and many detain and deport migrants, often withholding or withdrawing TB treatment [35]. Punitive migration laws drive the most vulnerable away from TB diagnosis and treatment—violating fundamental human rights relating to discrimination and healthcare access. This can be true of both international migrants and internally, as seen in China’s hukou registration system that makes many migrants ineligible for subsidized healthcare, resulting in lower access to TB care, poor treatment outcomes, and drug resistance.[40]

The Southern African Development Community (SADC) offers a better model, ensuring migrants access to care. Implementation remains challenging, but the SADC’s “Harmonised Minimum Standards for the Prevention, Treatment and Management of Tuberculosis” aim to improve referral systems and continuity of care for individuals who cross borders [41]. Countries should replicate this model, replacing failed efforts to erect borders with systems ensuring treatment access and respect for migrant rights.

## From law reform to action

The COVID-19 pandemic has laid bare the challenges public health authorities face in building trust and securing participation in public health efforts—including trust from migrant populations and trust from populations concerned that their basic rights will not be protected under intrusive public health measures. The TB response requires more than scaling up rapid diagnosis, treatment, and research into new therapeutic countermeasures. In a time when the global TB community is putting greater emphasis on activities like active case finding and contact tracing, public health actors are pushed increasingly into private spaces of the home, family, and workplace. Law reform is essential to safeguard human rights and proscribe discrimination. Without shifts in the legal environment, major gaps in TB diagnosis and treatment may well continue. This brief review shows that laws in HBCs too often fail to protect dignity and rights in the ways long shown to encourage engagement with the health system. Expanded focus on finding the missing TB cases could also result in stigma against vulnerable populations like migrants. Extant migration laws that restrict or ban travel and immigration for those with TB—rather than a humane cross-border TB response—will only fuel international spread of a virulent, often drug-resistant, infection. Fundamental human rights, expected in other areas of public life and in the health system, are often missing for people with TB. Building legal capacities for rights-based law reform (trained legislative drafters, human rights lawyers, and a compassionate judiciary) should be a high priority of UN agencies and member states.

## Abbreviations

HBC: high TB-burden countries
HLM: high-level meeting
SADC: Southern African Development Community
TB: tuberculosis
WHO: World Health Organization
